# Effects of polymorphisms in *ERCC1*, *ASE-1 *and *RAI *on the risk of colorectal carcinomas and adenomas: a case control study

**DOI:** 10.1186/1471-2407-6-175

**Published:** 2006-07-03

**Authors:** Camilla F Skjelbred, Mona Sæbø, Bjørn A Nexø, Håkan Wallin, Inger-Lise Hansteen, Ulla Vogel, Elin H Kure

**Affiliations:** 1 Department of Laboratory Medicine, Section of Medical Genetics, Telemark Hospital, N-3710 Skien, Norway; 2 Telemark University College, Faculty of Arts and Sciences, Department of Environmental and Health Studies, Hallvard Eikas plass, N-3800 Bø i Telemark, Norway; 3 Institute of Human Genetics, University of Aarhus, Aarhus, Denmark; 4 National Institute of Occupational Health, Copenhagen, Denmark; 5 Department of Pathology, Ullevaal University Hospital, Oslo, Norway

## Abstract

**Background:**

The risk of sporadic colorectal cancer is mainly associated with lifestyle factors and may be modulated by several genetic factors of low penetrance. Genetic variants represented by single nucleotide polymorphisms in genes encoding key players in the adenoma carcinoma sequence may contribute to variation in susceptibility to colorectal cancer. In this study, we aimed to evaluate whether the recently identified haplotype encompassing genes of DNA repair and apoptosis, is associated with increased risk of colorectal adenomas and carcinomas.

**Methods:**

We used a case-control study design (156 carcinomas, 981 adenomas and 399 controls) to test the association between polymorphisms in the chromosomal region 19q13.2-3, encompassing the genes *ERCC1*, *ASE-1 *and *RAI*, and risk of colorectal adenomas and carcinomas in a Norwegian cohort. Odds ratio (OR) and 95% confidence interval (CI) were estimated by binary logistic regression model adjusting for age and gender.

**Results:**

The *ASE-1 *polymorphism was associated with an increased risk of adenomas, OR of 1.39 (95% CI 1.06–1.81), which upon stratification was apparent among women only, OR of 1.66 (95% CI 1.15–2.39). The *RAI *polymorphism showed a trend towards risk reduction for both adenomas (OR of 0.70, 95% CI 0.49–1.01) and carcinomas (OR of 0.49, 95% CI 0.21–1.13) among women, although not significant. Women who were homozygous carriers of the high risk haplotype had an increased risk of colorectal cancer, OR of 2.19 (95% CI 0.95–5.04) compared to all non-carriers although the estimate was not statistically significant.

**Conclusion:**

We found no evidence that the studied polymorphisms were associated with risk of adenomas or colorectal cancer among men, but we found weak indications that the chromosomal region may influence risk of colorectal cancer and adenoma development in women.

## Background

Colorectal cancer (CRC) still is one of the leading causes of cancer deaths in developed countries and adenomas are the predominant precursors of this disease [1].

The risk of sporadic CRC is mainly associated with lifestyle factors and may be modulated by several genetic factors of low penetrance [2,3]. Genetic variants represented by single nucleotide polymorphisms (SNPs) in genes encoding key players in the adenoma carcinoma sequence may contribute to variation in susceptibility to CRC. To better understand what kind of impact, and at what stage in cancer progression certain SNPs contribute to CRC susceptibility, implementing cases with both pre-malignant and malignant neoplasia in case-control studies may be valuable.

Many published candidate gene association studies have assessed cancer risk by examining a single SNP per gene or a single locus at a time analysis approach. It has been suggested that use of haplotypes in association studies may have increased power over single-allele studies [4]. Haplotype analyses take into account a number of tightly linked markers, which are much more informative than individual markers. Haplotype analyses can identify unique chromosomal segments likely to harbor disease predisposing genes. Recently, a region in chromosome 19q13.2-3 encompassing the genes *ERCC1*, *ASE-1 *and *RAI *was identified as a high-risk haplotype, defined as homozygous carriers of a haplotype consisting of *ERCC1 *Asn118Asn^A^, *ASE-1 *G-21A^G ^and *RAI *IVS1 A4364G^A^. This haplotype consisting of three marker SNPs, is shown to be strongly associated with increased risk of skin cancer [5], breast cancer [6] and lung cancer [7] when compared to persons that are not homozygous carriers of the haplotype. No association has been observed with testicular cancer [8]. It is possible that this high-risk haplotype would be associated with risk of colorectal cancer because it seems to be a marker of general mechanisms affecting several cancer forms. The region of chromosome 19 seems to be involved in the balance between growth and elimination of DNA damage and unwanted cells [8].

The *ERCC1 *gene encodes a subunit of the nucleotide excision repair complex required for the incision step of nucleotide excision repair (NER) [9,10]. Since the ERCC1 protein is essential for NER and influence genomic instability, polymorphisms in *ERCC1 *may play a role in carcinogenesis.

The *ASE-1 *gene (Anti Sense ERCC1) also called *CD3EAP *(CD3e antigen, epsilon polypeptide associated protein), encodes a nucleolar protein localized to fibrillar centers of the nucleolus and to the nucleolus organizer region of mitotic chromosomes [11]. The mouse homolog of ASE-1 is part of the RNA polymerase I complex [12].

The gene *RAI *(RelA-associated Inhibitor) also called *iASSP *and *PPP1R13L*, is an inhibitor of the RelA subunit of the transcription factor NF-κB [13]. The NF-κB plays a pivotal role in the inflammatory response and apoptosis. It is, therefore, likely that polymorphisms affecting the level or activity of the RelA-associated inhibitor RAI would influence the availability of RelA, and thus modify regulation of apoptosis.

In the present paper, we aimed to evaluate whether the recently identified haplotype encompassing genes of DNA repair and apoptosis, is associated with increased risk of colorectal adenomas and carcinomas in a homogenous Norwegian cohort. In addition we investigated whether polymorphisms in these genes alone contributed to colorectal adenoma and carcinoma risk. To the best of our knowledge this high-risk haplotype has not previously been studied in relation to colorectal adenomas or carcinomas. Studying colorectal adenomas in addition to colorectal cancer may give information about the risk factors in the earlier stages of carcinogenesis

## Methods

The KAM cohort (Kolorektal cancer, arv og miljø) is based on the screening group of the Norwegian Colorectal Cancer Prevention study (The NORCCAP study) in the county of Telemark [14], and a series of CRC cases operated on at Telemark Hospital (Skien) and Ulleval University Hospital (Oslo). Those invited to participate in the NORCCAP study were 20,780 men and women, age 50–64 years old, drawn by randomization from the population registry in Oslo (urban) and the county of Telemark (mixed urban and rural). They were invited to have a flexible sigmoidoscopy screening examination with or without (1:1) an additional faecal occult blood test (FOBT). Seven hundred and seventy-seven individuals were excluded according to exclusion criteria. The overall attendance rate was 65%. The ID number for the NORCCAP study at ClinicalTrials.gov is I [NCT00119912] [15]. The screened cases in the NORCCAP study selected for colonoscopy were invited to participate in the KAM study (1044 cases agreed). During a limited period of time, after the screening study of NORCCAP was well established, controls (polyp free by sigmoidoscopy) were invited to participate in the KAM study (400 controls agreed). Written consent was obtained from all the participants. The KAM cohort is based on an ethnic homogenous group of Norwegian origin.

The KAM biobank consists of blood and tissue samples from 160 CRC cases (16 identified in the NORCCAP screening group and 144 from hospitals), 1044 individuals identified with adenomas in the large intestine (991 high- and low-risk adenomas, 53 hyperplastic polyps) and 400 controls, defined as individuals with normal findings at flexible sigmoidoscopy screening. Adenoma cases and controls were all drawn from the NORCCAP study. All of the participants completed a questionnaire on demographics, health status, dietary, and smoking habits, alcohol consumption, physical exercise and occupation. The questionnaire contained information on a family history of adenomas and carcinomas, and the included cases and controls had no known personal history of genetic predisposition. The adenocarcinomas were collected prior to chemo- or radiotherapy treatment. Two specialist histopathologists examined the histology of the adenomas independently in order to determine the tumor stage as mild, moderate or severe dysplasia. They reached the same conclusion in all cases. The KAM study is approved by the Regional Ethics Committee and the Data Inspectorate.

In the present study we analyzed available blood samples from 156 cases with adenocarcinoma, 981 cases with adenomas, (i.e. 227 high-risk and 754 low-risk adenomas) and 399 controls. The 53 hyperplastic polyps were not included in the analysis. Diminished numbers of available cases in the KAM biobank and analyzed cases are due to loss of samples during preparation and/or lack of information from questionnaires. A high-risk adenoma is defined as an adenoma measuring = 10 mm in diameter and/or with villous components and/or showing severe dysplasia [14]. The distribution of gender and age among controls and cases with colonic carcinomas and adenomas are shown in Table 1.

Genomic DNA was isolated from blood samples according to standard procedures [16] with minor modifications as previously described [17].

Genotypes were determined on an ABI 7500 using endpoint readings or on a light cycler (Roche Molecular Biochemicals, Mannheim, Germany). *ERCC1 *Asn118Asn (rs#3177700) was determined on a light cycler as described [6].*ASE-1 *G-21A (rs#967591) was determined using the primers 5'-TCT GCA ACC TGG TGC GAG-3', 5'-CCT TTC TCC TTC CAC CAA CG-3' and the probes G-allele: 5'-FAM- AGG GTT GCC TGA GGT GTG GGT CC-BHQ, A-allele: Yakima Yellow- AGG GTT ACC TGA GGT GTG GGT CC-BHQ-3'. The reactions were run for 40 cycles at 15 s at 94°C, 60 s at 62°C.*RAI *IVS1 A4364G (rs#1970764) was determined as described [8]. In general, 10 μl reactions contained ca. 50 ng DNA, 5 μl Mastermix (Applied Biosystems, Birkerød, Denmark), 100 nM of each probe and 900 nM of each primer. Controls were included in each run, and 10 % of the samples were retyped with identical results.

MiniTab Statistical Software, Release 13.1 Xtra (Minitab Inc.) and SPSS (Statistical Packages for the Social Sciences) 12.0.1 for Windows were used for the statistic calculations. Odds ratios (OR) and 95 % confidence intervals (CI) were calculated using binary logistic regression to assess the relationship between each polymorphism and the colorectal adenoma or carcinoma cases. When, estimating OR for carriers of the haplotype the comparison group was all the other remaining individuals, individuals who were not homozygous carriers of the high-risk haplotype. We have previously shown that neither known heterozygotes nor possible heterozygotes are at increased risk of breast cancer. This strongly suggests that the haplotype is detecting a recessive marker. We have therefore chosen only to look at homozygous carriers' vs. the rest. All data are adjusted for age and gender.

Smoking dose and alcohol intake were also included in the adjustment in the logistic regression analyses, but the results did not differ from those adjusted only for age and gender. Including smoking and alcohol parameters resulted in lower numbers of cases and controls due to missing data in the questionnaires. Due to this we choose to use the data adjusted only for age and gender.

With 156 CRC cases and 399 controls we have a 74 % chance (at the 5 % level) to detect an OR of 2 for the *ERCC1 *polymorphism (allele frequency 0.41) and a 92 % chance for the *ASE-1 *polymorphism (allele frequency 0.18) and the *RAI *polymorphism (allele frequency 0.20).

## Results

The genotypic distribution of the three polymorphisms in the *RAI*, *ASE-1 *and *ERCC1 *genes for both cases and controls are shown in Table 2 [see Additional file 1]. The genotype distributions for the studied polymorphisms were all in Hardy-Weinberg equilibrium (*ERCC1 *Asn118Asn p = 0.945, *ASE-1 *G-21A p = 0.757 and *RAI *IVS1 A4364G p = 0.673), and the distributions of the alleles are in agreement with those found in other Scandinavian populations [7,8]. Table 2 [see Additional file 1] also presents the estimates of relative risk associated with the three polymorphisms as well as the predefined haplotype.

The *RAI *polymorphism showed a trend towards risk reduction for both adenomas and carcinomas in women. The risk estimate was lowest but not statistically significant for carcinoma. For adenomas the result was borderline statistically significant, OR of 0.70 (95% CI 0.49–1.01).

The *ASE-1 *polymorphism was associated with an increased risk of adenomas, OR of 1.39 (95% CI 1.06–1.81), which upon stratification was apparent among women only, OR of 1.66 (95% CI 1.15–2.39). No association was found for carcinoma cases.

There was no significant association between the *ERCC1 *polymorphism and risk of colorectal adenomas and carcinomas.

Women who were homozygous carriers of the high risk haplotype had an increased risk of colorectal cancer, OR of 2.19 (95% CI 0.95–5.04) compared to all non-carriers although the estimate was not statistically significant. Among men an inverse association between the high-risk haplotype and colorectal cancer was found, although the association was not statistically significant. No association was found with adenoma cases.

We have also tested the gene-environment interaction between the haplotype and the three polymorphisms, and cigarette smoking and alcohol intake, but they showed no significant association with colorectal adenoma or carcinoma (results not shown).

## Discussion

In this case-control study we investigated whether carriers of a specific haplotype, encompassing genes of DNA repair and apoptosis located on chromosome 19q13.2-3, were at increased risk of colorectal adenomas and carcinomas. In addition we investigated whether polymorphisms in these genes alone contributed to colorectal adenoma and carcinoma risk.

The high-risk haplotype has previously been shown to be associated with increased risk in young or middle-aged persons. For basal cell carcinoma, effects were only seen for the age group below 50 years [5,18,19]. For lung cancer and breast cancer, an effect was observed for women in the age interval 50–55 years [6,7]. We detected no association (prior to gender stratification) among patients with colorectal adenomas or carcinomas for the high-risk haplotype. It is possible that at least the colorectal cancer patients in this material are too old to allow a detection of an effect. In accordance with this, no effect of the high risk haplotype was observed for basal cell carcinoma patients who were older than 50 years of age [20]. However, we did detect an effect in women and an inversely effect in men after stratifying for gender, in relation to CRC cases, although not significant. There are indications that effect of the haplotype may vary between the sexes because the haplotype was associated with increased risk of breast cancer [6] and not with risk of testis cancer [8]. In lung cancer Vogel *et al*. [21] have found statistically significant differences between sexes. Women who are homozygous carriers of the haplotype are at increased risk of lung cancer compared to men.

The polymorphism in *RAI *is shown to be the strongest determinant of the haplotype [6,21]. RAI was discovered as an inhibitor of RelA (a subunit of NF-κB) and is thus presumably involved in the control of transcription [13], but its function is poorly understood. RAI is involved in control of apoptosis, and over-expression of RAI has been shown to suppress apoptosis induced by cisplatin or UV light [22]. Up regulation of RAI expression has been observed in cancer cells [22,23]. Previous data suggest that the predefined haplotype is associated with defective apoptosis [21]. It is therefore likely that the haplotype is linked to a functional polymorphism in *RAI *which increases the protein level of RAI, resulting in a defective apoptosis trigger. It has previously been observed that the polymorphism in *RAI *and the high risk haplotype may be associated with cancer risk in women only [6-8]. For the polymorphism in *RAI*, we have consistently found protective effect of the variant allele. For lung cancer, however, it was found that the variant allele was protective among women (AG: RR= 0.58, CI 0.33–1.03, GG: RR = 0.12, CI 0.01–1.04, p trend 0.007), while no protective effect was observed for men (AG: 1.79, CI 1.12–2.87, GG: RR = 0.51, CI 0.13–2.05, Ole Raaschou-Nielsen and Ulla Vogel, unpublished results). Here, we see the same pattern. Among women, carriers of the variant allele of *RAI *IVS1 A4364G were at reduced risk of both colorectal cancer (OR= 0.49, CI 0.21–1.13) and adenomas (OR = 0.70, CI 0.49–1.01). Vogel *et al*. [24] have shown that the RAI mRNA level is 41 % higher in lymphocytes from women compared to men, which may indicate that a gender difference could be due to variation in transcription levels which subsequently result in differences in protein level. A possible explanation for our gender specific findings could be that women who are homozygous carriers of the haplotype have a defective apoptosis trigger (high levels of RAI). Colon cells with excessive DNA damage do not undergo apoptosis but survive with the potential of subsequent malignant transformation.

Very little is known about the function of ASE-1. Whitehead *et al*. [11] identified the ASE-1 gene product as a nucleolar protein by screening a HeLa cDNA library with a human autoimmune serum. The mouse Pol I-associated factor PAF49 shows striking homology to the human nucleolar protein ASE-1. Immunolocalization analysis revealed that PAF49 accumulated in the nucleolus of growing cells but dispersed to nucleoplasma in growth-arrested cells. These results strongly suggest that PAF49/ASE-1 plays an important role in rRNA transcription [12]. Involvement in the immune function of this gene product has also been suggested [25]. We found that the observed risk estimate was highest among the heterozygote carriers, which may suggest that we detect an effect of another, linked polymorphism. The fact that we observe an effect for adenomas but not for colorectal cancer makes a chance finding possible. Another explanation for the observed association in the adenoma group only could be that the polymorphism may play a role in regression of adenomas rather than in progression to cancer.

The CRC cases and controls have not been matched by age which may affect the results in a study. However, the controls were recruited from the same cohort as the adenoma and carcinoma cases. The adenoma cases and controls are from 50 to 64 years of age. Further, our controls have been screened and found polyp free and the risk of any of them having colon cancer at the time of inclusion is not very likely.

Taking the number of statistical tests performed and the p-values we cannot exclude that our findings are due to chance. The relatively small numbers of the CRC cases limited our power to detect and association with carcinomas. However, the findings for *RAI *are consistent with previous findings [5-8].

## Conclusion

The high-risk haplotype is not associated with increased risk of adenomas or colorectal cancer prior to gender stratification. When stratifying for gender, we did detect an effect in women and an inverse effect in men in relation to CRC cases, although not significant. Of the high-risk haplotype markers, the *ASE-1 *polymorphism was associated with an increased risk of adenomas in women. The *RAI *polymorphism showed a trend towards risk reduction for both adenomas and carcinomas in women, although not significant. There was no significant association between the *ERCC1 *polymorphism and risk of colorectal adenomas and carcinomas. In conclusion we found no evidence that the studied polymorphisms were associated with risk of adenomas or colorectal cancer among men, but we found weak indications that the chromosomal region may influence risk of colorectal cancer and adenoma development in women.

## Competing interests

The author(s) declare that they have no competing interests.

## Authors' contributions

CFS prepared samples for analysis and contributed with the data analysis. She prepared the first draft of the paper. MS prepared samples for analysis and contributed with the data analysis. She also contributed to the manuscript. ILH participated in collection and quality control of the questionnaires. UBV, HW and BAN genotyped the DNA samples. EHK brought the idea of the KAM study and organized it. All authors discussed the results, contributed to interpretation of the results and the final manuscript.

## Pre-publication history

The pre-publication history for this paper can be accessed here:



## Supplementary Material

Additional file 1 **Table 2: Distributions of *RAI, ASE-1 *and *ERCC1 *genotypes and high-risk haplotype, and development of colorectal carcinomas and adenomas**. The table presents the genotypic distribution of the three polymorphisms in the *RAI*, *ASE-1*, and *ERCC1 *genes for both cases and controls, and the estimates of relative risk associated with the three polymorphisms as well as the predefined haplotype. Click here for file

## Figures and Tables

**Table 1:** Distributions of gender and age among controls and cases with colorectal carcinomas and adenomas.

	Controls	Carcinomas	Adenomas, all	High-risk adenomas	Low-risk adenomas
	No (%)	No (%)	No (%)	No (%)	No (%)
*No. of subjects*	399	156	981	227	754
*Sex*^a^					
Male	157 (39.3)	86 (55.1)	601 (61.3)	150 (66.1)	451 (59.8)
Female	242 (60.7)	70 (44.9)	380 (38.7)	77 (33.9)	303 (40.2)
*Age*^b^					
Mean (SD)	54.2 (3.3)	67.9 (11.2)	57.3 (3.7)	57.3 (3.5)	57.3 (3.8)

^a ^There are significant differences in the number of males and females in the control group and the case groups, p < 10^-4 ^(test for comparison of two proportions).

^b ^There are significant differences in age among the control group and the case groups, p < 10^-4 ^(Mann-Whitney test).
