# Supplementary material for: Effects of polymorphisms in ERCC1, ASE-1 and RAI on the risk of colorectal carcinomas and adenomas: a case control study
Source: BMC Cancer. 2006 Jul 3;6:175. doi: 10.1186/1471-2407-6-175 (PMC1533843; doi:10.1186/1471-2407-6-175)
Supplement: Additional file 1 — Table 2: Distributions of RAI, ASE-1 and ERCC1 genotypes and high-risk haplotype, and development of colorectal carcinomas and adenomas. The table presents the genotypic distribution of the three polymorphisms in the RAI, ASE-1, and ERCC1 genes for both cases and controls, and the estimates of relative risk associated with the three polymorphisms as well as the predefined haplotype. [file 1471-2407-6-175-S1.pdf]

Table 2: Distributions of *RAI, ASE-1* and *ERCC1* genotypes and high-risk haplotype, and development of colorectal carcinomas and adenomas.

Genotypes	Controls	Carci-nomas	OR (95% CI)	p	Adenomas	OR (95% CI)	p	High-risk adenomas	OR (95% CI)	p	Low-risk adenomas	OR (95% CI)	p
***RAI* IVS1 A4364G**													
AA	253	105	1[a]		663	1[a]		152	1[a]		511	1[a]	
AG	133	50	0.82 (0.46-1.47)	0.511	286	0.90 (0.69-1.17)	0.430	65	0.86 (0.58-1.28)	0.460	221	0.89 (0.67-1.18)	0.406
GG	13	1	-	-	31	0.79 (0.39-1.59)	0.507	10	1.41 (0.58-3.46)	0.450	21	0.68 (0.32-1.45)	0.320
AG + GG	146	51	0.78 (0.44-1.40)	0.407	317	0.89 (0.68-1.16)	0.385	75	0.93 (0.63-1.36)	0.704	242	0.88 (0.67-1.17)	0.374
Missing					1						1		
***ASE-1* G-21A**													
GG	270	116	1[a]		612	1[a]		140	1[a]		472	1[a]	
GA	115	35	0.72 (0.38-1.36)	0.310	334	**1.41** (1.07-1.86)	0.014	80	**1.47** (1.10-4.60)	0.048	254	**1.37** (1.03-1.82)	0.032
AA	14	5	0.90 (0.19-4.31)	0.900	35	1.16 (0.59-2.26)	0.668	7	1.09 (0.40-2.95)	0.863	28	1.20 (0.59-2.40)	0.617
GA + AA	129	40	0.70 (0.38-1.29)	0.256	369	**1.39** (1.06-1.81)	0.017	87	**1.47** (1.00-2.17)	0.048	282	1.30 (0.98-1.72)	0.065
***ERCC1* Asn118Asn**													
AA	140	68	1[a]		353	1[a]		80	1[a]		273	1[a]	
AG	185	64	0.79 (0.43-1.44)	0.436	464	1.04 (0.79-1.38)	0.773	102	1.04 (0.70-1.55)	0.858	362	1.04 (0.78-1.39)	0.787
GG	66	21	1.37 (0.62-3.02)	0.438	147	0.93 (0.64-1.36)	0.716	40	1.19 (0.70-2.02)	0.512	107	0.89 (0.60-1.32)	0.556
AG + GG	251	85	0.88 (0.50-1.55)	0.652	611	1.03 (0.79-1.35)	0.836	142	1.15 (0.78-1.70)	0.473	469	0.98 (0.74-1.30)	0.902
Missing	8	3			17			5			12		
**Haplotype of the above high-risk[b]**													
No	305	114	1[a]		750	1[a]		179	1[a]		571	1[a]	
Yes	86	39	1.25 (0.65-2.41)	0.507	213	0.95 (0.69-1.30)	0.740	43	0.76 (0.48-1.20)	0.239	170	1.04 (0.75-1.44)	0.802
Missing	8	3			18			5			13		
<u>WOMEN</u>													
***RAI* IVS1 A4364G**													
AA	146	50	1[a]		264	1[a]							
AG + GG	96	20	0.49 (0.21-1.13)	0.093	115	0.70 (0.49-1.01)	0.057						
Missing					1								
***ASE-1* G-21A**													
GG	170	50	1[a]		225	1[a]							
GA + AA	72	20	0.65 (0.27-1.56)	0.334	155	**1.66** (1.15-2.39)	0.006						
***ERCC1* Asn118Asn**													
AA	87	29	1[a]		125	1[a]							
AG + GG	148	39	0.76 (0.35-1.65)	0.493	248	1.20 (0.83-1.73)	0.329						
Missing	7	2			7								

**Haplotype of the above high-risk**[b]							
No	184	47	1[a]		293	1[a]	
Yes	51	21	2.19 (0.95-5.04)	0.066	79	0.95 (0.62-1.45)	0.811
Missing	7	2			8		
MEN							
***RAI* IVS1 A4364G**							
AA	107	55	1[a]		399	1[a]	
AG + GG	50	31	1.30 (0.55-3.03)	0.548	202	1.17 (0.78-1.75)	0.446
Missing							
***ASE-1* G-21A**							
GG	99	66	1[a]		387	1[a]	
GA + AA	58	20	0.81 (0.34-1.95)	0.642	214	1.13 (0.76-1.67)	0.554
***ERCC1* Asn118Asn**							
AA	53	39	1[a]		228	1[a]	
AG + GG	103	46	1.07 (0.45-2.52)	0.881	363	0.87 (0.58-1.29)	0.478
Missing	1	1			10		
**Haplotype of the above high-risk**[b]							
No	121	67	1[a]		457	1[a]	
Yes	35	18	0.54 (0.17-1.64)	0.275	134	0.95 (0.60-1.50)	0.824
Missing	1	1			10		

[a] The genotype served as reference category

[b] Homozygotes for the haplotype *RAI* IVS1 A4364G[A] *ASE-1* G-21A[G] *ERCC1* Asn118Asn[A].
